# Investigating the causal relationship of lipid metabolism in polycystic ovary syndrome: a Mendelian randomization study on the regulatory role of 3-Hydroxybutyrate in gene expression

**DOI:** 10.3389/fmed.2026.1747593

**Published:** 2026-02-04

**Authors:** Jia Xu, Lan Li, Shuo Yang, Ping Li, Bing He, Ji-lin Kuang

**Affiliations:** 1 Department of Gynaecology and Obstetrics, The Second Affiliated Hospital of Hunan University of Chinese Medicine, Changsha, China; 2 College of Integrated Traditional Chinese and Western Medicine, Hunan University of Chinese Medicine, Changsha, China

**Keywords:** 3-hydroxybutyrate, gene expression, HDAC3, lipid metabolism, Mendelian randomization, metabolic dysfunction, polycystic ovary syndrome

## Abstract

**Introduction:**

Polycystic ovary syndrome (PCOS) is a prevalent endocrine disorder affecting 5–18% of reproductive-aged women, characterized by menstrual irregularities, hyperandrogenism, and polycystic ovarian morphology. Beyond its endocrine manifestations, PCOS involves significant metabolic dysfunction, particularly in lipid homeostasis. Elevated triglyceride levels are closely linked to insulin resistance and cardiovascular risk, suggesting a central role for lipid dysregulation in PCOS pathogenesis. However, traditional observational studies struggle to establish causal relationships due to confounding factors and reverse causality.

**Methods:**

To address these limitations, this study employed a two-sample Mendelian randomization (MR) design to assess the causal effects of lipid-related metabolites—specifically triglyceride-rich lipoprotein subclasses—on PCOS susceptibility. Furthermore, to elucidate potential biological mechanisms, we integrated the MR analysis with *in vitro* functional experiments, focusing on the role of ketone body metabolism and specifically 3-hydroxybutyrate (3-HB), a major circulating ketone body known to regulate gene expression via epigenetic modifications.

**Results:**

Our analysis identified a causal contribution of lipid-related metabolites to PCOS. notably, we demonstrated that 3-HB plays a critical role in the development of PCOS. Mechanistic investigations revealed that 3-HB contributes to metabolic and hormonal dysregulation primarily through the modulation of HDAC3 activity, linking ketone body metabolism directly to the disease phenotype.

**Conclusion:**

This study provides robust causal evidence linking lipid metabolism and ketone bodies to PCOS, moving beyond descriptive associations. by uncovering the specific pathway involving 3-HB and HDAC3, we highlight a novel molecular mechanism underlying PCOS pathogenesis. These findings suggest that targeting the 3-HB/HDAC3 axis could offer new strategies for therapeutic intervention in managing PCOS-related metabolic dysfunction.

## 1 Introduction

Polycystic Ovary Syndrome (PCOS) is one of the most common endocrine disorders worldwide, affecting approximately 5–18% of women of reproductive age (1). Its clinical features include menstrual irregularities, hyperandrogenism (such as hirsutism and acne), and polycystic ovaries (2, 3). Although the etiology of PCOS is not fully understood, an increasing number of studies have shown that PCOS is not only a manifestation of endocrine disorders but also involves metabolic abnormalities, especially alterations in lipid metabolism. Lipid metabolism disorders, particularly those related to triglycerides, have been confirmed as one of the main phenotypes of PCOS and are closely related to insulin resistance, obesity, and cardiovascular diseases in PCOS (4). Therefore, lipid metabolism may play a core role in the pathogenesis of PCOS.

Traditional observational studies often have difficulty in clearly establishing the relationship between metabolites and PCOS due to confounding factors, reverse causality, and potential biases (5, 6). Mendelian Randomization (MR), as a causal inference method based on genetic variations, can overcome these problems and has shown unique advantages in the study of metabolic diseases, especially PCOS (7). By identifying single nucleotide polymorphisms (SNPs) related to exposure factors (such as metabolites) from genome-wide association studies (GWAS), MR can effectively avoid the confounding factors and reverse causality problems in traditional studies, thereby providing more accurate causal inferences.

Although previous studies have shown a certain association between lipid metabolism and PCOS, most of them have remained at the level of phenotypic association analysis, lacking in-depth causal inference and mechanism exploration (8–10). Therefore, this study aims to reveal the causal relationship between lipid metabolites, especially triglyceride-related lipoprotein particles and PCOS, through two-sample MR analysis, and further explore the potential mechanisms of these metabolites in the occurrence of PCOS. In addition, ketone body metabolism, especially 3-hydroxybutyrate (3-HB), as a key intermediate product in energy metabolism, may also regulate ovarian function through epigenetic mechanisms and be closely related to the occurrence of PCOS. Therefore, the goal of this study is to reveal the causal role of metabolites and ketone body metabolism in the occurrence of PCOS through MR analysis, and further verify the biological effects of 3-HB through *in vitro* experiments, providing new perspectives and potential targets for the treatment of PCOS.

### 1.1 Research design

To explore the relationship between metabolites and polycystic ovary syndrome (PCOS), we designed a two-sample Mendelian randomization (MR) study (11). MR studies need to meet the following assumptions (12): (1) a strong and robust correlation between instrumental variables (IVs) and exposure (association assumption); (2) independence of IVs from confounding factors that affect the relationship between exposure and outcome (independence assumption); (3) gene variations only affect the outcome through the exposure factor and not through other means (exclusion restriction assumption).

### 1.2 Data sources

All data on exposure and outcome in this study were obtained from the IEU website.^1^ All the data used have been published in public databases and do not require additional ethical approval. The MR analysis we conducted used single nucleotide polymorphisms (SNPs) related to metabolites as exposure factors and PCOS as the outcome. Metabolite data were from the article by Karjalainen et al. (13), and PCOS data were from the EBI database (ID: ebi-a-GCST90044902).

### 1.3 Selection of instrumental variables

To fulfill the first assumption of MR, SNPs closely related to the exposure factor were selected (*p* < 5.0 × 10^−8^, *r*^2^ = 0.001, genetic distance = 10,000 kb). To fulfill the second assumption of MR, we queried the Phenoscanner database^2^ to ensure that the selected SNPs were not related to known confounding factors. Finally, the F-statistic was calculated to assess whether there was weak instrument bias in the selected instrumental variables (14). An *F* > 10 indicates no weak instrument bias, further supporting the association assumption. The formula for calculating F is: F = [R^2^/(1 − R^2^)] × [(N − K −1)/K], where N is the sample size of the exposure factor, K is the number of instrumental variables, and R^2^ is the proportion of variation in the exposure factor explained by the instrumental variables (15).

### 1.4 Mendelian randomization analysis

All MR analyses in this study were conducted using the “TwoSampleMR,” “MendelianRandomization,” and “ggplot2” packages in R language (4.3.3). The inverse variance weighted method (IVW) was used as the primary analysis method, and the weighted median, MR-Egger regression, simple model, and weighted model were used as auxiliary analysis methods.

### 1.5 Sensitivity analysis

The Cochrane Q value and MR-Egger intercept were used to assess heterogeneity and horizontal pleiotropy, respectively (16). Leave-one-out analysis was used to detect whether the association between exposure and outcome was mainly influenced by a single SNP. Sensitivity analysis was visualized using scatter plots, funnel plots, and forest plots to demonstrate the robustness of the MR study. The odds ratio and its 95% confidence interval were used to quantitatively assess the potential causal relationship between the exposure factor and the outcome. All statistical tests were two-tailed, and *p* < 0.05 was considered statistically significant. The study results were reported in accordance with the STROBE-MR guidelines (17).

### 1.6 Experimental verification

#### 1.6.1 Materials

KGN human ovarian granulosa cell line (OriCell, Catalog No. H6-1301), fetal bovine serum (FBS), DMEM high glucose medium, 1% penicillin/streptomycin solution. HDAC3-specific inhibitor RGFP966 (MCE, Catalog No. HY-13909), 3-Hydroxybutyrate (MCE, Catalog No. HY-113378). Human Testosterone, T ELISA Kit (cusabio, Catalog No. CSB-E05099h), Human Estradiol, E2 ELISA Kit (cusabio, Catalog No. CSB-E05108h), SYBR qPCR SuperMix Plus (novoprotein, Catalog No. E096-01A), Plus All-in-one 1st Strand cDNA Synthesis SuperMix (novoprotein, Catalog No. E047-01B).

#### 1.6.2 Cell culture

Culture conditions: Cells were cultured in DMEM/F12 medium containing 10% fetal bovine serum (FBS) and 1% penicillin/streptomycin at 37 °C in a 5% CO₂ incubator. After the cells reached 80–90% confluence, they were divided into different treatment groups for processing.

#### 1.6.3 Experimental grouping

Control group: Only medium. 3-HB treatment group: Given 3-HB. 3-HB inhibition group: Treated with 3-HB -specific inhibitor (RGFP966) and 3-HB simultaneously.

#### 1.6.4 Cell toxicity assay

After the cells reached 80–90% confluence, KGN cells were seeded into 96-well plates at a density of 1 × 104 cells per well. In the 3-HB treatment group, 3-HB was added at predetermined concentrations (0.5, 1, 2, 3, 4, 5 mM), and CCK8 reagent was added after 24 h. In the HDAC3 inhibition group, RGFP966 was added at predetermined concentrations (10, 20, 40, 80, 160 nM), and CCK8 reagent was added after 24 h.

#### 1.6.5 ELISA

After drug treatment, the supernatants of each group were collected, and the contents of testosterone and estradiol were detected according to the kit instructions.

#### 1.6.6 qRT-PCR

Total RNA was extracted from tissue samples using Trizol, and the RNA samples were reverse transcribed into cDNA using the HiFiScript cDNA First Strand Synthesis Kit. qRT-PCR was performed using the CFX ConnectTM Real-Time PCR System (Bio-Rad, USA). The primer sequences of the genes are shown in Table 1. The relative mRNA expression of the target genes in each sample was calculated using the 2^−△△CT^ method.

**Table 1:** Primers sequence.

Gene	Forward (5′–3′)	Reverse (5′–3′)
GAPDH	CATGAGAAGTATGACAACAGCCT	AGTCCTTCCACGATACCAAAGT
FSHR	TCTGTCACTGCTCTAACAGGG	TGCACCTTTTTGGATGACTCG
LHCGR	TTCCAAGGGATGAATAACGAGTCT	TGCATGGCTTTGTACTTCTTCAA
CYP19A1	AACAACTCGACCCTTCTTTATG	TTTGAGGGATTCAGCACAG
HDAC3	TGATGACCAGAGTTACAAGCAC	GGGCAACATTTCGGACAG
IRS1	TTGAGAATGTGTGGCTGAGG	TCCTTGACCAAATCCAGGTC
StAR	CAGACTTCGGGAACATGCCT	CCCTTGAGGTCGATGCTGAG
CYP11A1	CACTCCTCAAAGCCAGCATCA	ACGAAGCACCAGGTCATTCAC
CYP17A1	GGGCGGCCTCAAATGG	CAGCGAAGGCGAAGGCGATACCCTTA
HSD3B2	TCTCAGATGACACGCCTCAC	GGGCTGAGTAGGAAGCTCAC
HSD17B13	CCTACTTGGAGTCGTTGGTGA	CCAATATGCTCTGTCGTTTTGC

#### 1.6.7 Statistical analysis

Statistical analysis was performed using Graphpad prism 5.0 software. Experimental data are presented as mean ± standard deviation (SD). Comparisons between two groups were made using one-way analysis of variance (One-way ANOVA).

## 2 Results

### 2.1 Instrumental variable characteristics

After applying stringent selection criteria—including genome-wide significance screening (*p* < 1 × 10^−5^), elimination of variants in linkage disequilibrium (LD pruning), effect allele alignment, MR-PRESSO testing, and F-statistic evaluation—the final set of SNPs used as instrumental variables (IVs) all exhibited *F*-values exceeding 10. This indicates that each selected IV has a strong and statistically robust association with the respective gut microbiota traits, minimizing the risk of weak instrument bias.

### 2.2 Genetic evidence for metabolite effects on polycystic ovary syndrome

**Figure 1:**
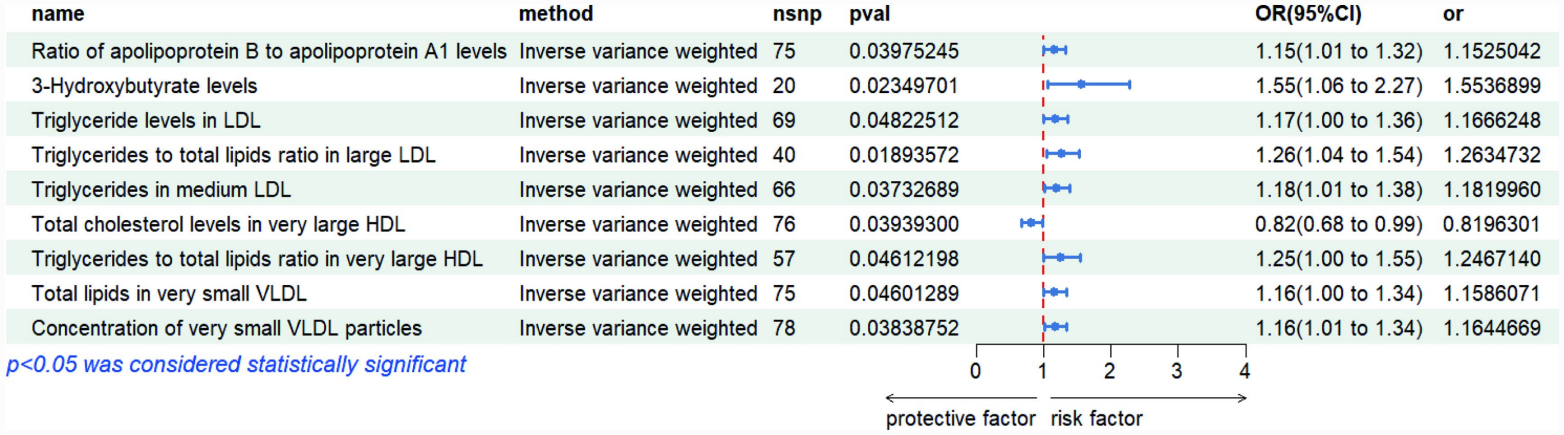
Risk plots of various metabolites IVW results.

**Figure 2:**
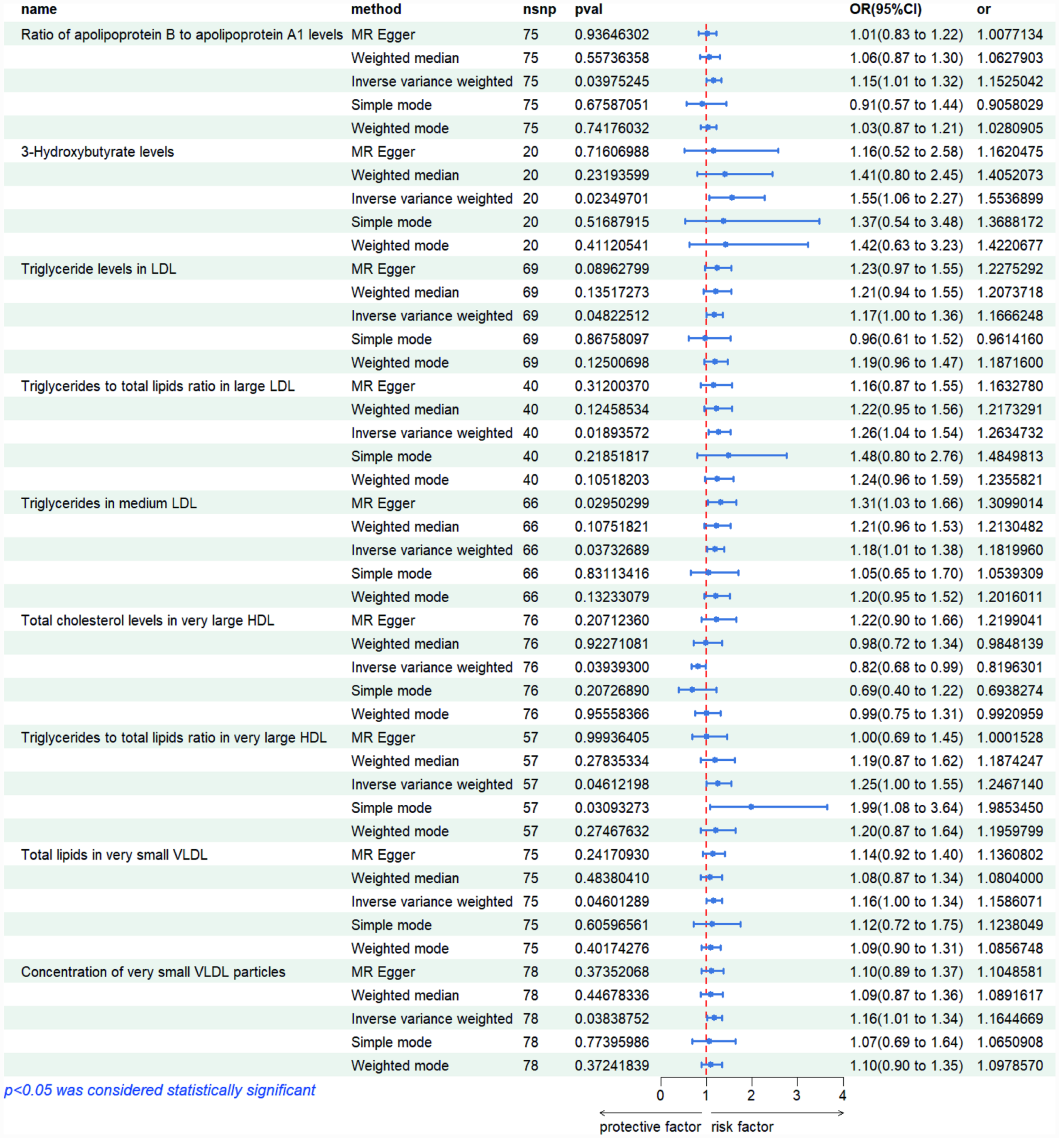
Risk graphs of all results for various metabolites.

Mendelian randomization analysis revealed several lipid-related metabolites with significant causal associations with polycystic ovary syndrome (PCOS). Notably, triglyceride measures within low-density lipoprotein (LDL) and very low-density lipoprotein (VLDL) subclasses demonstrated consistent positive effects: higher triglyceride content in LDL particles (IVW OR = 1.167, *p* = 0.048), increased ratio of triglycerides to total lipids in large LDL (IVW OR = 1.263, *p* = 0.019), and elevated triglyceride levels in medium-sized LDL (IVW OR = 1.182, *p* = 0.037) were all linked to greater disease risk. Similarly, both total lipid amount (IVW OR = 1.159, *p* = 0.046) and particle number (IVW OR = 1.164, *p* = 0.038) in extremely small VLDL subfractions were positively associated with PCOS. Furthermore, elevated ratios of apolipoprotein B to A1 (IVW OR = 1.153, *p* = 0.040) and increased levels of 3-hydroxybutyrate (IVW OR = 1.554, *p* = 0.023) suggested enhanced risk, whereas higher concentrations of cholesterol in very large high-density lipoprotein (HDL) particles were found to be protective (IVW OR = 0.820, *p* = 0.039). Collectively, these findings highlight the potential role of dysregulated lipid metabolism—particularly involving triglyceride-enriched lipoproteins and ketone body pathways—in the etiology of PCOS. These genetically informed results provide compelling support for the involvement of lipid homeostasis in PCOS pathogenesis, offering novel insights into metabolic mechanisms underlying disease susceptibility. The detailed outcomes are illustrated in Figures 1, 2.

### 2.3 Sensitivity analysis

**Figure 3:**
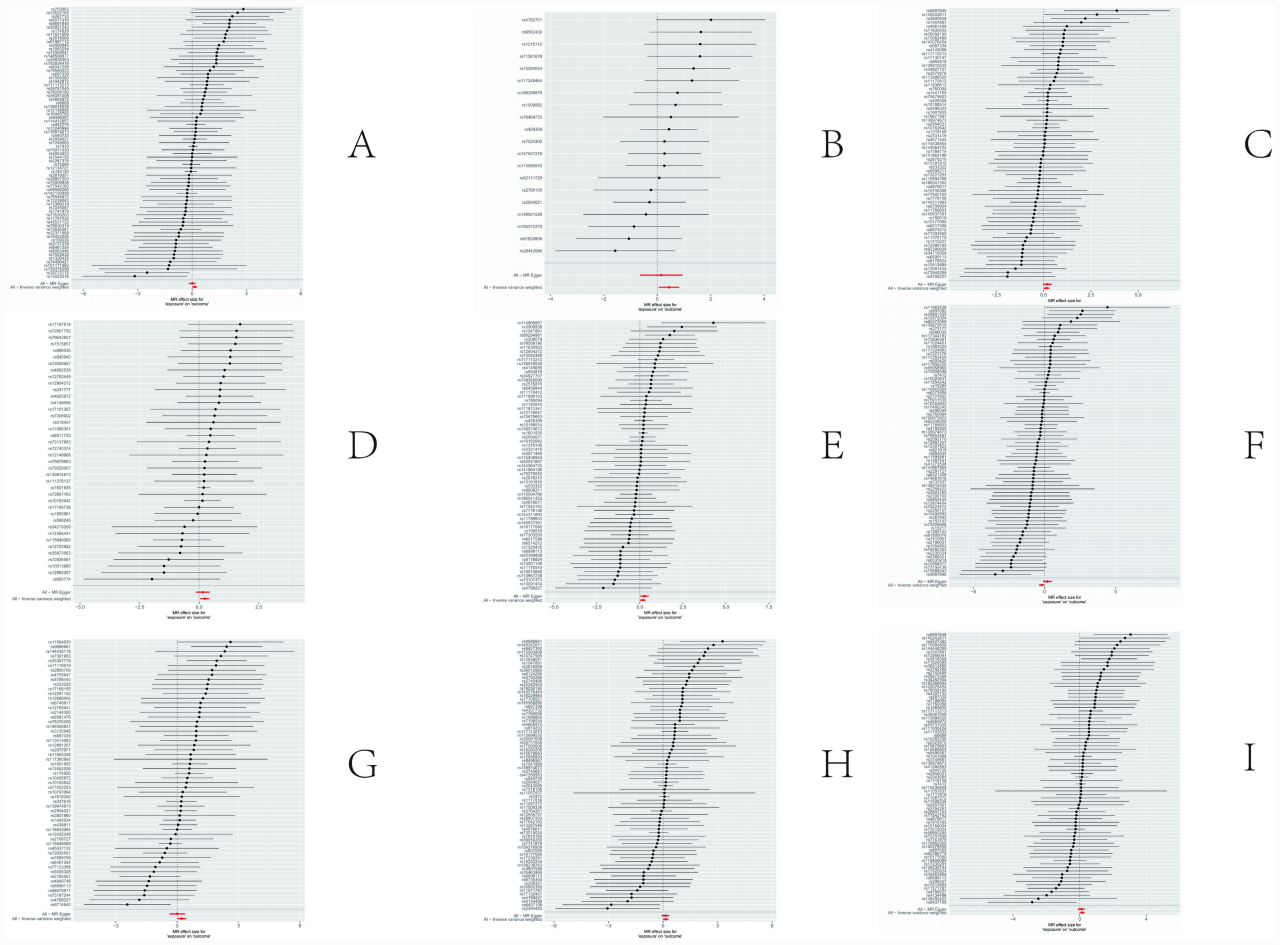
Results of sensitivity analysis-forest. **(A)** Ratio of apolipoprotien B to apolipoprotien A1 levels; **(B)** 3-hydroxybutyrate levels; **(C)** triglyceride levels in LDL; **(D)** triglycerides to total lipids ratio in large LDL; **(E)** triglyceride in medium LDL; **(F)** total cholesterol levels in very large HDL; **(G)** triglycerides to total lipids ratio in very large HDL; **(H)** total lipids in very small VLDL; **(I)** concentration of very small VLDL particles.

**Figure 4:**
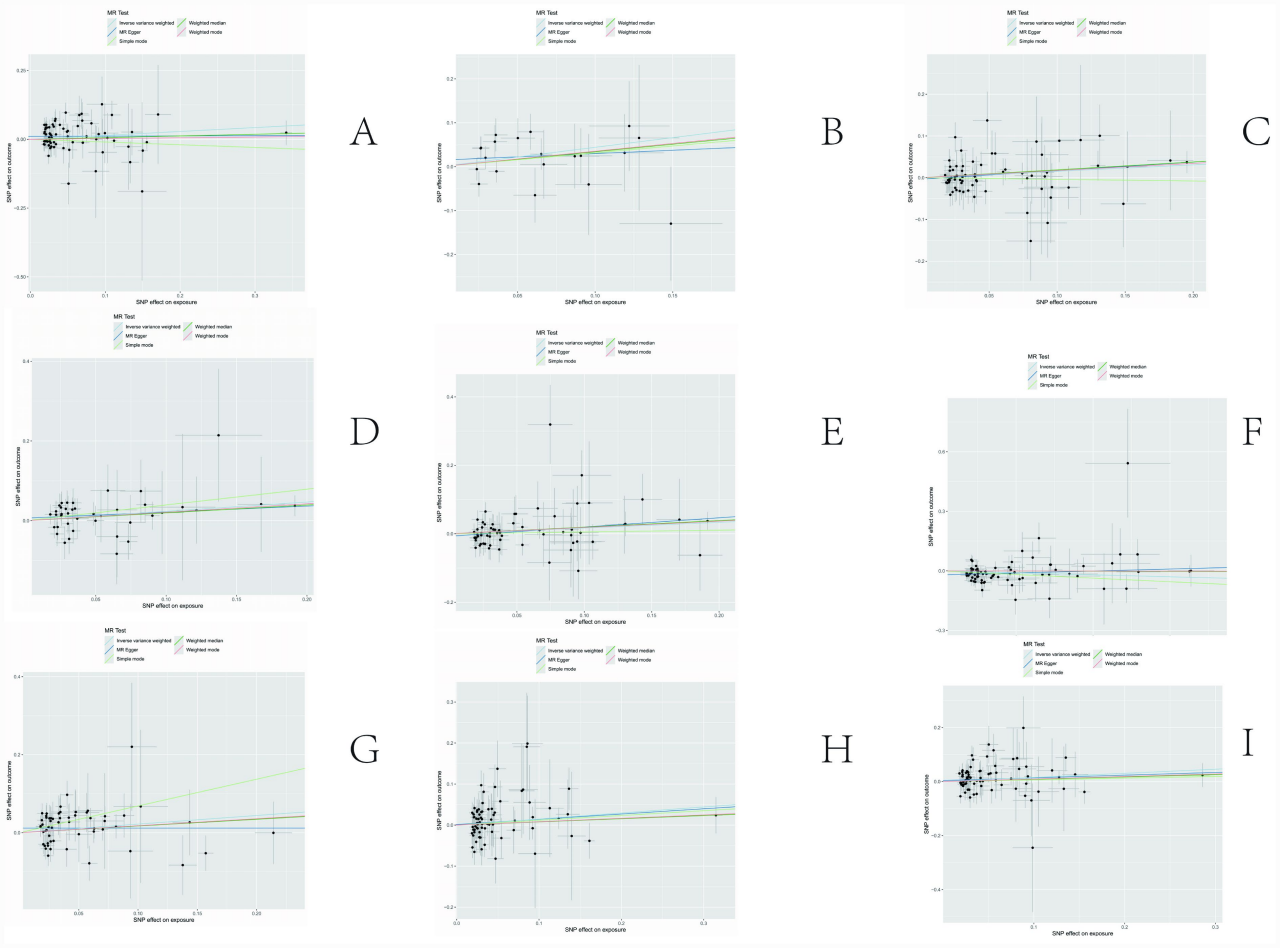
Results of sensitivity analysis-scatter. **(A)** Ratio of apolipoprotien B to apolipoprotien A1 levels; **(B)** 3-hydroxybutyrate levels; **(C)** triglyceride levels in LDL; **(D)** triglycerides to total lipids ratio in large LDL; **(E)** triglyceride in medium LDL; **(F)** total cholesterol levels in very large HDL; **(G)** triglycerides to total lipids ratio in very large HDL; **(H)** total lipids in very small VLDL; **(I)** concentration of very small VLDL particles.

**Figure 5:**
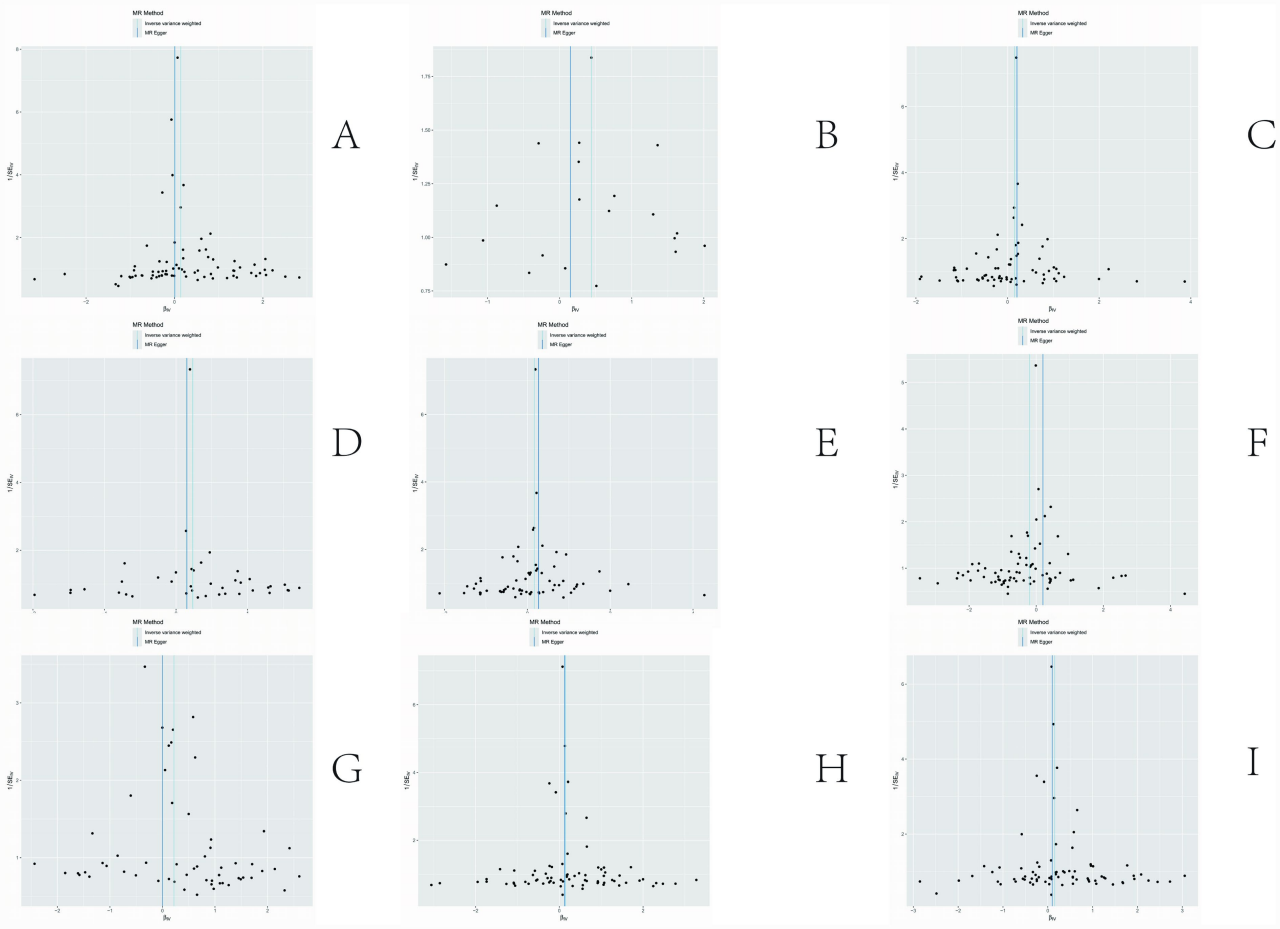
Results of sensitivity analysis-funnelplot. **(A)** Ratio of apolipoprotien B to apolipoprotien A1 levels; **(B)** 3-hydroxybutyrate levels; **(C)** triglyceride levels in LDL; **(D)** triglycerides to total lipids ratio in large LDL; **(E)** triglyceride in medium LDL; **(F)** total cholesterol levels in very large HDL; **(G)** triglycerides to total lipids ratio in very large HDL; **(H)** total lipids in very small VLDL; **(I)** concentration of very small VLDL particles.

The sensitivity analyses provided further validation of the robustness of the primary findings. Results obtained from multiple Mendelian randomization approaches—such as the weighted median and MR-Egger methods—were highly aligned with those derived from the inverse variance weighted method, showing no major discrepancies. The MR-Egger regression intercept test revealed no evidence of significant horizontal pleiotropy (*p* > 0.05), suggesting that the genetic variants primarily affected the outcome via the exposure of interest, thereby minimizing concerns about pleiotropy-induced bias. Although Cochran’s Q test indicated the presence of heterogeneity for certain exposures, the observed associations remained statistically significant under the random-effects model, implying that heterogeneity did not produce spurious results. Furthermore, the leave-one-out analysis demonstrated that no individual SNP disproportionately influenced the overall causal estimate, confirming that the findings were not driven by a single outlying genetic variant. Overall, these sensitivity assessments collectively reinforce the credibility and stability of the main analytical outcomes, lending strong support to the study’s core conclusions. The corresponding results are presented in Figures 3–5.

### 2.4 Analysis of metabolite target sites

Based on the above analysis, we found that an elevated level of 3-Hydroxybutyrate increases the risk of polycystic ovary syndrome, and its main target site is Histone deacetylase 3. Therefore, we speculate that Histone deacetylase 3 may be related to the pathogenesis of polycystic ovary syndrome. To further verify the biological effect of 3-Hydroxybutyrate, we used the KGN human ovarian granulosa cell line *in vitro*.

### 2.5 Cytotoxicity assay results

**Figure 6:**
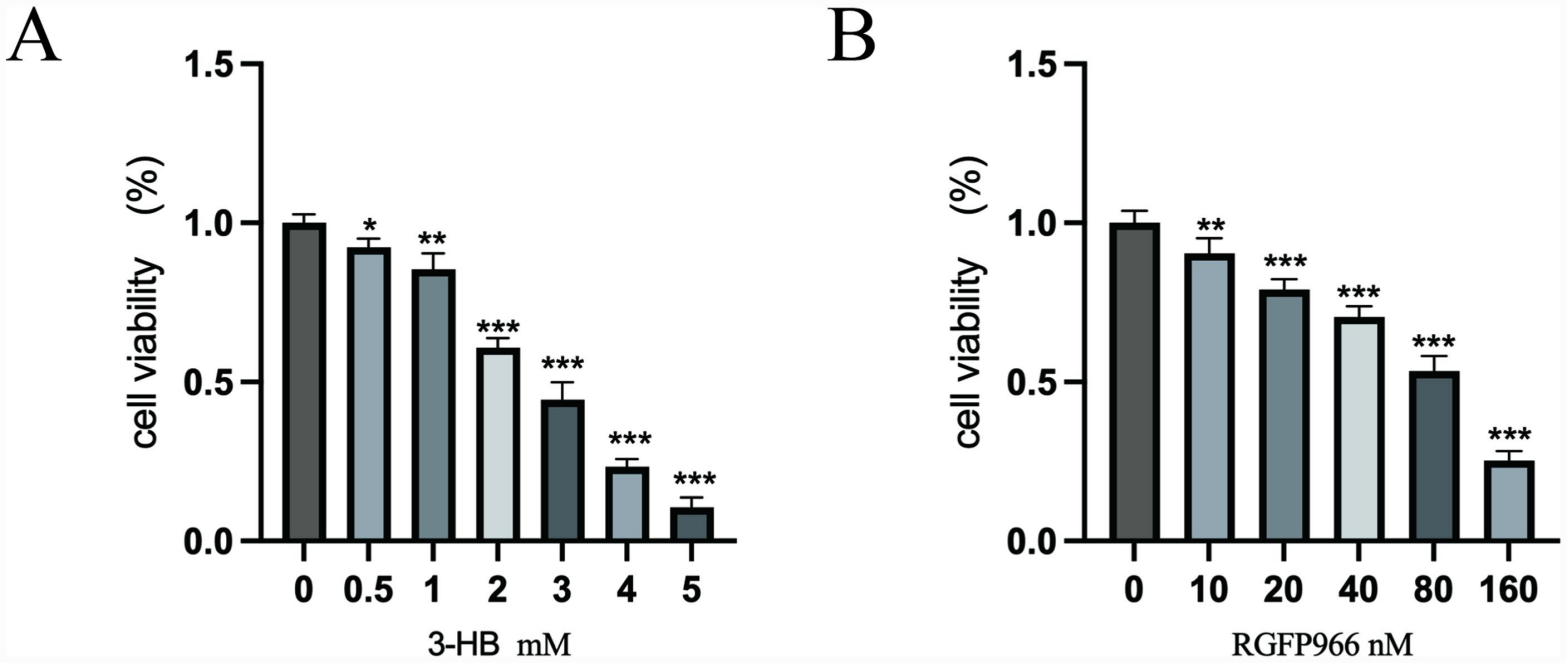
**(A)** 3-HB; **(B)** RGFP966. *p < 0.05, **p < 0.01, ***p < 0.001.

The CCK8 assay results indicated that 1 mM 3-HB had a minor effect on the viability of KGN cells within 24 h; similarly, 40 nM RGFP966 (HDAC3 inhibition) had a minor impact on the viability of KGN cells within 24 h. Subsequently, 1 mM 3-HB and 40 nM RGFP966 were selected for the experiment (Figure 6).

### 2.6 Measurement of testosterone and estradiol levels in cell culture supernatants

**Figure 7:**
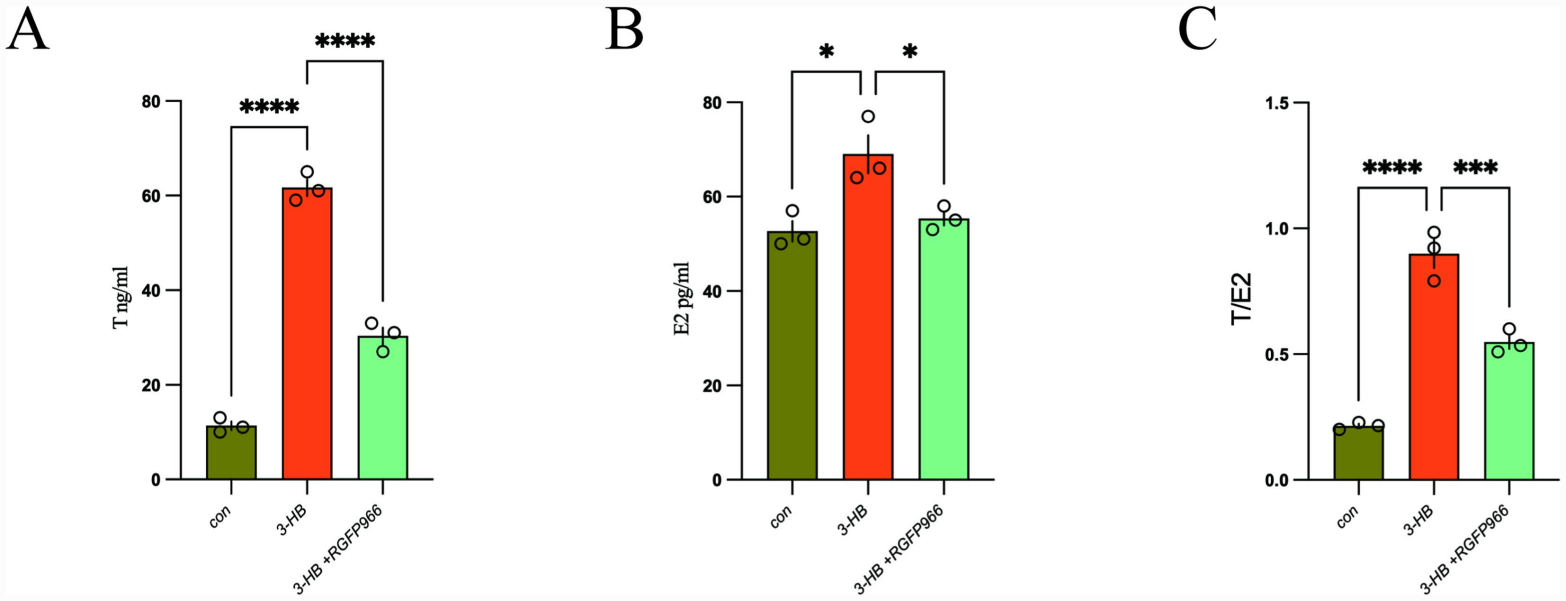
**(A)** Testosterone; **(B)** estrogen; **(C)** testosterone/estrogen ratio. *p < 0.05, ***p < 0.001, ****p < 0.0001.

The primary pathological of PCOS involves hyperandrogenism and disrupted estrogen homeostasis. ELISA analyses of cell culture supernatants revealed that 3-HB induces dysregulation of testosterone and estradiol levels. Furthermore, pharmacological blockade of 3-HB alleviates this hormonal imbalance between testosterone and estradiol (Figure 7).

### 2.7 The results of the qRT-PCR experiment

**Figure 8:**
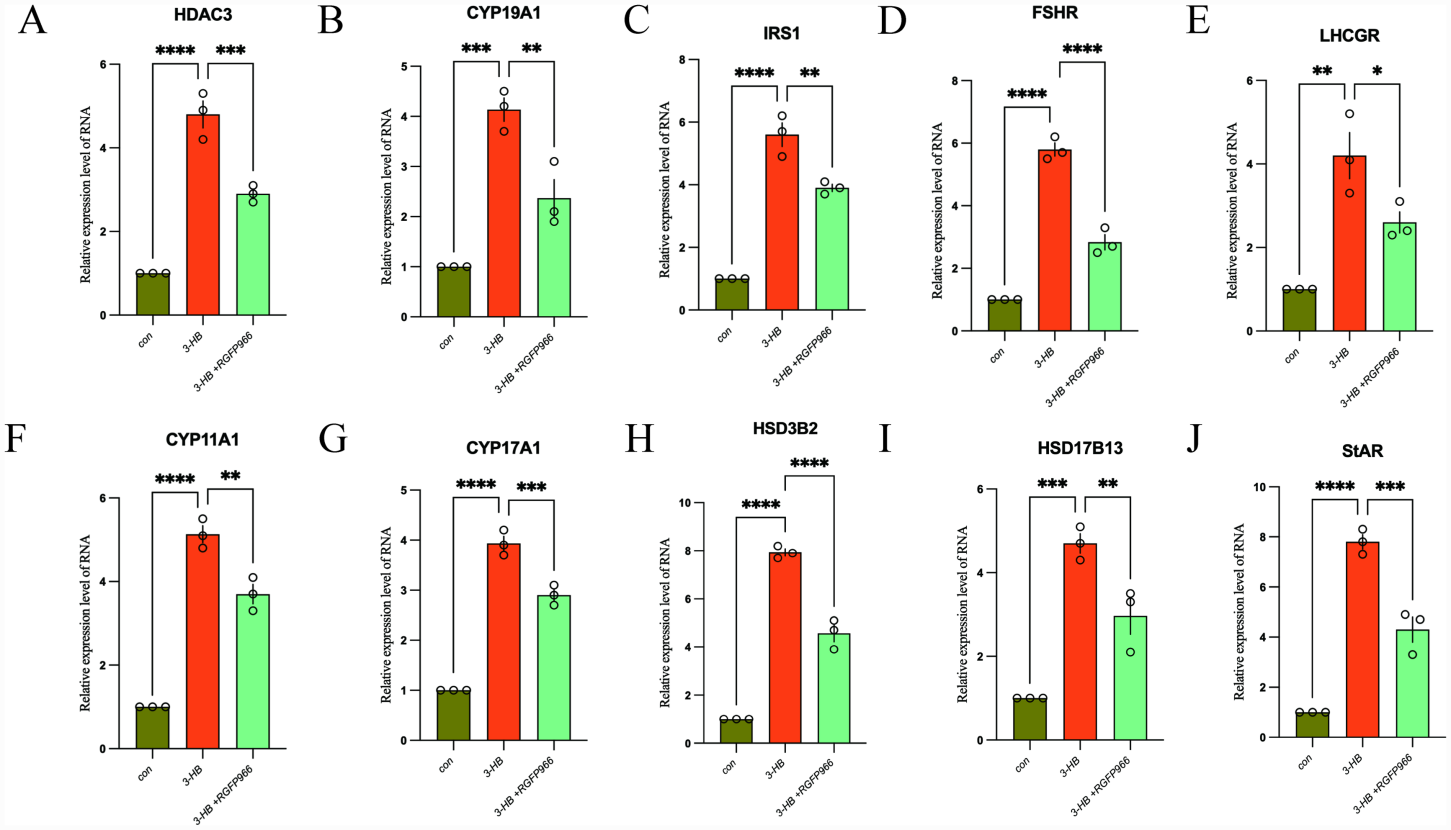
The expression changes of HDAC3 **(A)**, CYP19A1 **(B)**, IRS1 **(C)**, FSHR **(D)**, LHCGR **(E)**, CYP11A1 **(F)**, CYP17A1 **(G)**, HSD3B2 **(H)**, HSD17B13 **(I)**, and StAR **(J)** genes. *p < 0.05, **p < 0.01, ***p < 0.001, ****p < 0.0001.

Using qRT-PCR, we demonstrated that 3-HB significantly up-regulates the transcriptional levels of 10 genes—HDAC3, CYP19A1, IRS1, FSHR, LHCGR, StAR, CYP11A1, CYP17A, HSD3B2, and HSD17B13. Furthermore, pharmacological inhibition of 3-HB leads to a partial but consistent down-regulation in the expression of these genes (Figure 8).

## 3 Discussion

This study explored the potential role of 3-Hydroxybutyrate (3-HB) in the pathophysiology of Polycystic Ovary Syndrome (PCOS), combining Mendelian Randomization (MR) approaches with *in vitro* cellular assays. A central focus was placed on the involvement of Histone deacetylase 3 (HDAC3) as a mediating pathway. The results reveal a causal association between elevated 3-HB levels and increased PCOS susceptibility, highlighting HDAC3 as a key player in this molecular interplay.

### 3.1 Insights from Mendelian randomization analysis

The MR analysis demonstrated a statistically significant link between higher circulating 3-HB concentrations and an elevated risk of developing PCOS. Notably, 3-HB showed consistent positive associations with lipid-related metabolites, particularly triglycerides present in low-density lipoprotein (LDL) and very-low-density lipoprotein (VLDL) particles. These observations align with existing evidence suggesting that disturbances in lipid metabolism—especially those involving triglyceride-rich lipoproteins—and altered ketone body homeostasis may contribute to PCOS etiology (18, 19). Additionally, we identified a correlation between 3-HB and the apolipoprotein B/A1 ratio, indicating a multifaceted influence of 3-HB on metabolic and cardiovascular risk factors associated with PCOS. Sensitivity analyses confirmed the robustness of these findings, minimizing concerns about horizontal pleiotropy and reinforcing the validity of our conclusions (20).

### 3.2 Impact of 3-HB on gene expression in PCOS-related pathways

To further elucidate the biological mechanisms, we conducted *in vitro* experiments assessing how 3-HB modulates the expression of genes implicated in PCOS. Our data indicate that 3-HB exerts substantial regulatory effects on several critical genes involved in endocrine function, insulin signaling, and metabolic regulation.

A marked increase in HDAC3 mRNA levels was observed following 3-HB exposure. As a core epigenetic enzyme, HDAC3 regulates gene transcription by removing acetyl groups from histones and other proteins, thereby influencing diverse cellular processes (21). In the context of PCOS, HDAC3 may serve as a mediator of ovarian dysfunction and insulin resistance through epigenetic reprogramming. This positions HDAC3 as a pivotal factor in 3-HB-driven pathogenic pathways, warranting deeper investigation (22).

Moreover, treatment with 3-HB led to increased expression of FSHR (follicle-stimulating hormone receptor), LHCGR (luteinizing hormone receptor), and CYP19A1 (aromatase)—genes essential for gonadal hormone production and follicular development. Dysregulation of these receptors is commonly linked to hyperandrogenism, disrupted ovulation, and impaired folliculogenesis in PCOS (22, 23). Given that FSHR and LHCGR govern follicular maturation and steroidogenesis, while CYP19A1 controls estrogen biosynthesis, their altered expression under 3-HB influence may amplify hormonal imbalances characteristic of PCOS.

Additionally, 3-HB induced upregulation of IRS1 (insulin receptor substrate 1), a crucial component of the insulin signaling cascade closely tied to insulin resistance (24). Although enhanced IRS1 expression might initially appear beneficial for insulin sensitivity, in the context of PCOS, such changes could paradoxically disrupt downstream signaling, contributing to metabolic dysregulation. This suggests that 3-HB may indirectly worsen insulin resistance despite apparent activation of early insulin pathway elements.

Collectively, these findings suggest that 3-HB influences multiple genetic pathways relevant to PCOS pathogenesis via HDAC3-mediated regulation. By altering the expression of genes involved in steroidogenesis, insulin action, and ovarian physiology, 3-HB may drive key clinical features of PCOS, including menstrual irregularities, hyperandrogenemia, and metabolic disturbances (25, 26).

### 3.3 Role of HDAC3 in epigenetic regulation

HDAC3 plays a fundamental role in chromatin remodeling and transcriptional control through histone deacetylation. In this study, its upregulation was directly correlated with 3-HB treatment. Importantly, pharmacological inhibition of HDAC3 significantly reduced the effects of 3-HB on target gene expression, indicating that HDAC3 activity is functionally required for 3-HB’s regulatory actions. This implies that HDAC3 acts as a central node in the network connecting 3-HB to PCOS-related molecular alterations, particularly those affecting hormonal balance and metabolic function (27, 28).

These results position HDAC3 not only as a potential contributor to PCOS progression but also as a critical effector in the 3-HB signaling axis. Through modulation of gene networks governing insulin response, steroid hormone synthesis, and cellular energy metabolism, HDAC3 may accelerate disease development in individuals with elevated 3-HB levels (29, 30).

### 3.4 Proposed mechanistic framework linking 3-HB and PCOS

Integrating the genetic and functional evidence, we propose that 3-HB contributes to PCOS development primarily through HDAC3-dependent epigenetic regulation. By altering the expression of genes involved in sex hormone biosynthesis and insulin signal transduction, 3-HB may promote hyperandrogenism and estrogen deficiency. Concurrently, its influence on insulin-related genes may exacerbate metabolic impairments commonly seen in PCOS. Together, these pathways highlight 3-HB as a biologically active metabolite with significant implications for both reproductive and metabolic health in PCOS (31, 32). Targeting the 3-HB–HDAC3 axis may therefore represent a novel therapeutic strategy for managing or even preventing PCOS progression (33, 34).

### 3.5 Limitations and directions for future research

While this study provides compelling evidence linking 3-HB to PCOS through MR and experimental models, certain limitations must be acknowledged. First, the findings are based solely on cell culture systems without validation in human tissue samples or animal models. Future studies should incorporate clinical cohorts and *in vivo* experiments to confirm these mechanisms and assess translational potential. Second, although HDAC3 was identified as a major mediator, it is likely that 3-HB affects PCOS through additional epigenetic regulators or alternative signaling cascades. Further exploration into other possible pathways—such as DNA methylation, non-coding RNAs, or mitochondrial function—could provide a more comprehensive understanding of 3-HB’s role in metabolic-endocrine crosstalk.

In summary, this work uncovers a novel mechanism whereby 3-HB modulates PCOS-associated gene networks via HDAC3 activation, offering new insights into the metabolic underpinnings of PCOS. HDAC3 emerges as a promising therapeutic target, paving the way for innovative interventions aimed at disrupting the pathological loop between ketone body metabolism and reproductive dysfunction in PCOS.

## Data Availability

The datasets presented in this study can be found in online repositories. The names of the repository/repositories and accession number(s) can be found at: https://gwas.mrcieu.ac.uk/.

## Footnotes

1
https://gwas.mrcieu.ac.uk/

2
http://www.phenoscanner.medschl.cam.ac.uk/
